# A Novel Technique Using Paretic Superior Rectus as a Globe Suspender for Monocular Elevation Deficiency

**DOI:** 10.7759/cureus.46365

**Published:** 2023-10-02

**Authors:** Fatima AlGhazal, Waleed Khayyat, Saleh AlMesfer, Abdulaziz Awad, Gorka Sesma

**Affiliations:** 1 Pediatric Ophthalmology & Strabismus Division, King Khaled Eye Specialist Hospital, Riyadh, SAU

**Keywords:** case report, inferior rectus recession, strabismus, knapp surgery, eye muscle transposition, elevation deficit, hypotropia, monocular elevation deficiency

## Abstract

Surgical innovations in strabismus provide opportunities to improve visual function, eye alignment, and cosmesis in rare pediatric ophthalmological conditions. Monocular elevation deficiency is a rare and multifactorial disease in which the affected eye is equally limited in terms of elevation during adduction and abduction.

We aimed to present a novel procedure for the treatment of acquired monocular elevation deficiency using the paretic superior rectus muscle as a globe suspender to resolve hypotropia. We report the case of an eight-year-old girl with left eyelid ptosis and hypotropia two months after draining a left orbital abscess. Left inferior rectus muscle recession was performed at five years, with residual left hypotropia. Ophthalmological examination revealed a best-corrected visual acuity of 20/20 OD and 20/100 OS. Severe left eyelid ptosis and poor levator function were also observed. Extraocular motility showed left hypotropia of 40 prism diopters with the left superior rectus muscle under action (-4) in the adduction and abduction positions.

A force duction test negative for restrictions on the inferior rectus muscle was performed intraoperatively. To reduce the risks of the Knapp procedure, the left superior rectus muscle was split into medial and temporal halves. Double-armed sutures were secured in half, and the halves were detached from the sclera. The medial and temporal halves were reattached anteriorly to the medial and lateral rectus insertions, respectively. Eight weeks after surgery, the patient had nine prism diopters of hypotropia in the primary gaze. Ten weeks after surgery, there was no change in visual acuity. In the cover test, the patient exhibited residual left hypotropia of nine prism diopters with a restriction (-4) of elevation in adduction and abduction. The parents were pleased with the satisfactory cosmetic outcomes, and postoperative clinical photographs of the patient showed improved hypotropia and persistent minimal elevation of the left eye during adduction and abduction.

Superior rectus muscle splitting and vertical transposition to the medial and lateral rectus could be safer and simpler alternatives to the Knapp procedure and may offer a lower risk of anterior segment ischemia. Further studies are required to confirm these findings.

## Introduction

Surgical innovations in strabismus provide opportunities to improve visual function, eye alignment, and cosmesis in rare pediatric ophthalmological conditions. One such condition, monocular elevation deficiency (MED), formerly known as double elevator palsy, is a rare and multifactorial disease in which the affected eye is equally limited in terms of elevation during adduction and abduction [1]. In addition to hypotropia, these patients often exhibit pseudo-ptosis and eyelid ptosis. Disease onset can be congenital or acquired, and causes include supranuclear palsy, primary superior rectal paresis, primary inferior rectal restriction, cerebrovascular disorders, sarcoidosis, syphilis, tumors, and trauma [2,3].

MED is divided into the following three categories based on its pathogenesis: restriction of the inferior rectus muscle, insufficient innervation of the elevator muscles, and a combination of restriction and deficiency [4]. Surgery is recommended if there is severe ocular deviation or diplopia in the primary gaze, significantly abnormal head posture, or deviation-induced amblyopia [5]. The appropriate surgical procedure depends on the forced duction test (FDT) of the inferior rectus muscle [6]. If the FDT result for elevation is positive, an inferior rectus muscle recession is performed; if it is nonrestrictive, Knapp’s procedure or its modifications are the preferred approach [7,8]. These procedures may improve hypotropia by an average of 38 prism diopters (PD) with favorable results [9] but are difficult, intricate, and carry the risk of anterior segment ischemia (ASI) if the inferior rectus muscle has previously undergone an operation [10]. Contralateral superior rectus recession is an alternative treatment, particularly in patients with residual hypotropia. However, the ethical concerns regarding surgery in healthy eyes should be considered.

To date, the horizontal rectus tendon has been transposed superiorly in its entirety or in part. Lateral or inferior transposition of the superior rectus muscle is not seen as a viable alternative, most likely because of the paretic nature of the muscle, creating a knowledge gap regarding this possibility [7-9].

We hypothesized that two aspects could justify this surgical technique as an option. First, the basic characteristic of all skeletal muscle is that stretching increases both passive and active tension to a predetermined optimal length [11,12]. Second, if the divided superior rectus muscle is reattached at the level of the insertion of the medial and lateral rectus muscles, mechanical changes in the ensuing force vector of eye alignment act as a globe suspender, thereby improving hypotropia. Therefore, increased superior muscle tension and changes in the mechanical force vector owing to its new insertion could play a role in the rationale for this treatment.

We aim to report a case of acquired MED syndrome with good surgical outcomes after the superior rectus was split into the medial and lateral halves; the medial half was transposed to the insertion of the medial rectus muscle, and the lateral half was transposed to the insertion of the lateral rectus muscle to correct vertical deviation. To our knowledge, this unique surgical method has not been documented in the literature, and we believe that it may open the door for new treatment options and pave the way for new research on the optimal management of this rare disease.

## Technical report

This study was approved by the Research Department and the Institutional Review Board of King Khaled Eye Specialist Hospital (RP 23092-CR) and complied with the Declaration of Helsinki. Consent was obtained from both parents of the patient.

An eight-year-old girl presented to the pediatric ophthalmology clinic of a tertiary care hospital complaining of hypotropia and drooping of the left upper eyelid. She had undergone left orbital abscess drainage at the age of two months. She underwent a 7-mm left inferior rectus recession and a 12-mm levator muscle resection to correct hypotropia and ptosis elsewhere at the age of five years.

**Figure 1:**
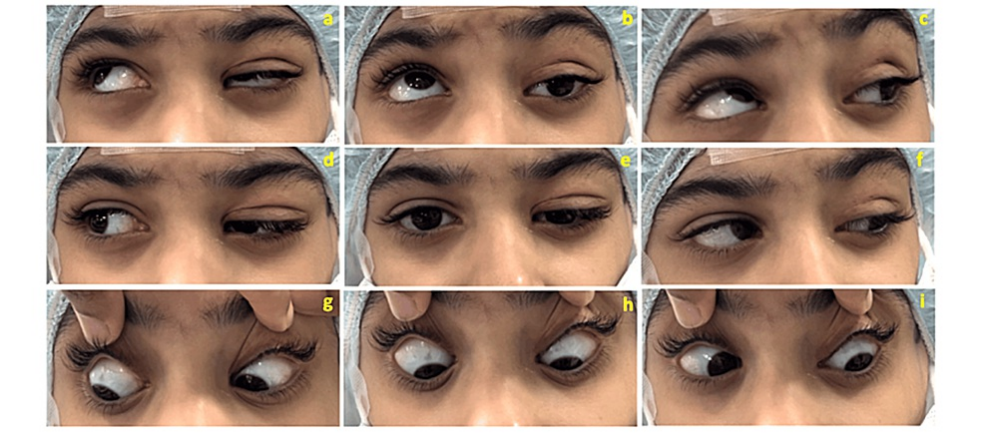
Preoperative clinical photographs showing eyelid ptosis and hypotropia with limited elevation in the left eye. Nine gaze photographs of an eight-year-old female with acquired monocular elevation deficiency of the left eye. (a-c) Dextroelevation and levoelevation gazes showing left eyelid ptosis and hypotropia with limited elevation. (d-f) Right, center, and left gazes demonstrating eyelid ptosis and left hypotropia of the left eye. (g-i) Dextrodepression, depression, and levodepression gazes confirming the normal function on depression of the left eye. These findings are consistent with superior rectus muscle paresis.

Upon examination, the patient was found to be reliant upon the right eye. The left eye had 3 mm eyelid ptosis that did not cover the pupil, and there was no abnormal head posture. The best-corrected visual acuity was 20/20 and 20/100 in the right and left eyes, respectively. Cycloplegic refraction was (sphere/cylinder × axis) OD: +3.00/-1.50 x 180; OS: + 4.00/-4.00 × 60. An extraocular motility examination revealed limited left eye elevation (-4) in the primary, adduction, and abduction positions. The cover test showed 45 PD hypotropia near and far from the primary deviation (Figure 1). The secondary deviation was right-sided hypertropy (70 PD). Anterior and posterior segment examinations did not reveal any abnormalities. Magnetic resonance imaging of the orbit and brain revealed unremarkable findings. The parents were informed of the left eye condition and treatment options and chose surgical intervention to correct the hypotropia.

**Figure 2:**
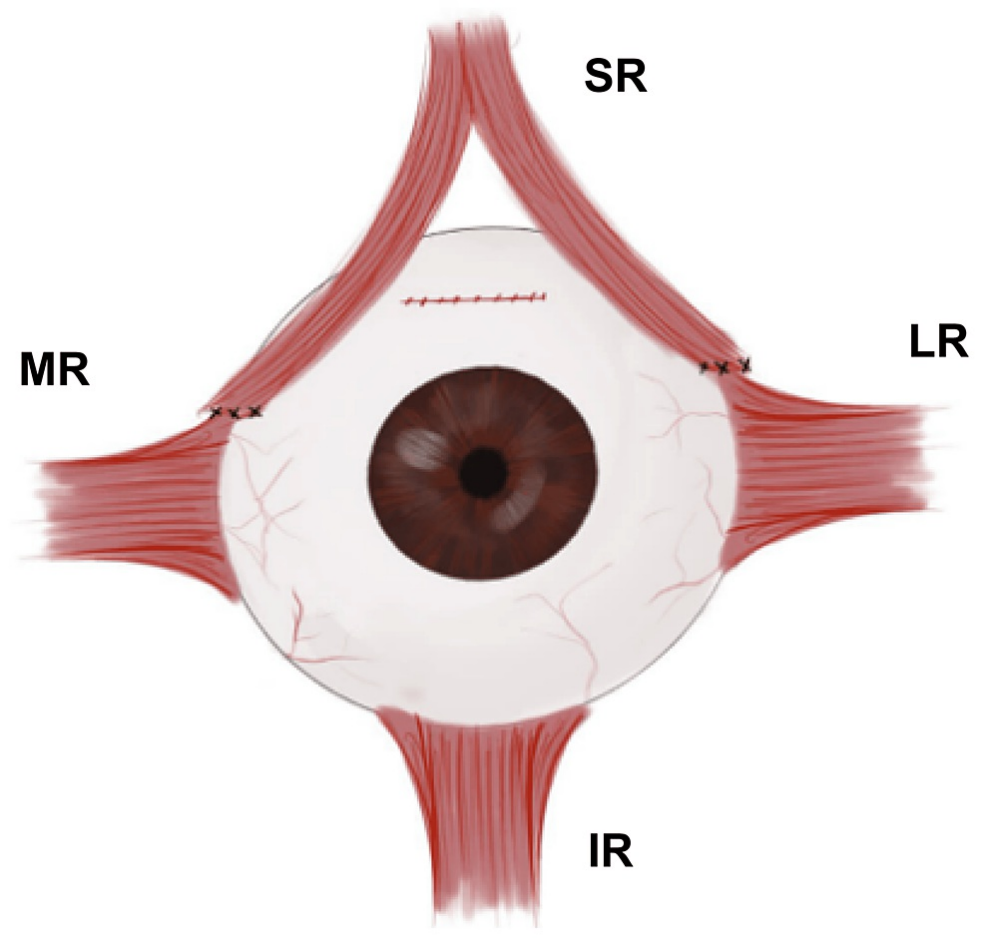
Sesma-AlGhazal procedure. Illustration of the split and transposed tendon site for the superior rectus muscle (SR) to the lateral rectus muscle (LR) and medial rectus muscle (MR) in the left eye.

The surgical procedure steps were as follows: under general anesthesia, we took prior antisepsis measures, properly draped the patient, and placed the eyelid speculum. Using two Thorpe corneal fixation forceps (Karl Storz SE, Baden-Wurttemberg, Germany) applied at the 3 and 9’clock positions in the corneal limbus, we performed an FDT in the left inferior rectus muscle, and no restriction was observed. We used Moody fixation forceps (Corzamedical Ophthalmology, Parsippany, NJ, USA) to track down and expose the superior quadrant of the eye by placing them on the superonasal limbus. To access the subtenon space, we made a conjunctival incision 8 mm from the limbus, opened the underlying Tenon capsule, and resected it. A nasal incision was successfully made, and the left superior rectus muscle was transected 10 mm into two halves, a medial and a temporal half, using 6-0 double-armed polyglactin sutures. We then removed the muscle from the sclera and hooked the medial rectus muscle, displacing it superiorly to create space for the attachment of the nasal half of the superior rectus, which we reattached 1 mm anterior to the medial rectus insertion. The lateral rectus muscle was hooked through a superotemporal incision and shifted upward, after which the superior rectus muscle was reattached to the sclera, located 1 mm above the lateral rectus insertion point (Figure 2). Following the procedure, we used 8-0 polyglactin sutures to close both conjunctival incisions. We treated the left eye for the next three weeks with an ophthalmic ointment containing neomycin, polymyxin B sulfates, and dexamethasone (Maxitrol, Alcon Labs Inc, Fort Worth, TX, USA).

**Figure 3:**
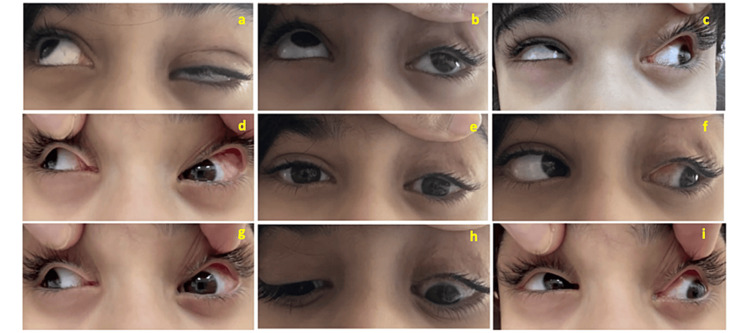
Postoperative clinical photographs of the patient showing improved hypotropia and persistent minimal elevation of the left eye during adduction and abduction. Nine gaze photographs of an eight-year-old female with acquired monocular elevation deficiency of the left eye. (a-c) Upright, upward, and upleft gazes showing left eyelid ptosis and improved left hypotropia with persistent limited elevation. (d-f) Right, center, and left gazes demonstrating eyelid ptosis and improved residual hypotropia of the left eye. (g-i) Downright, downward, and downleft gazes confirming limitation on levodepression.

Ten weeks after surgery, there was no change in visual acuity. In the cover test, the patient exhibited residual left hypotropia of 9 PD with a limitation (-4) of elevation in primary, adduction, and abduction. The parents were pleased with the satisfactory cosmetic outcomes. The patient with persistent eyelid ptosis was sent to the Oculoplastic Division for treatment and amblyopia patching therapy was initiated. Postoperative clinical photographs of the patient showed improved hypotropia and persistent minimal elevation of the left eye during adduction and abduction (Figure 3).

## Discussion

We describe a new surgical approach to correct hypotropia in a young girl with acquired MED. As the patient had previously undergone inferior rectus recession, this new method was used to prevent complications, including ASI, which may have occurred if two or more muscles were used for correction, as in the Knapp procedure [10].

Several techniques have been proposed to manage MED. Contralateral superior rectus muscle recession is a valid and efficient option, but the operative issue is controversial in terms of unnecessary risks to healthy eyes [6,13]. The current preferred choice for correction is the Knapp procedure when the FDT is negative [7]. Other corrective surgical methods include the enhanced Knapp procedure, superior transposition of a single horizontal muscle, the modified Nishida procedure, and recession and resection of the vertical muscles [9,13,14]. Most of these techniques focus on transferring the lateral and medial rectus to the superior rectus with relatively good results, and many of the strategies would require the use of two or more muscles in our patient, increasing the risk of complications, including ASI, especially after the inferior rectus muscle has been operated on. Most of these techniques have only been described in a small series, and their long-term success is yet to be reported. Furthermore, transposing the horizontal muscles superiorly, as in the preferred approach, may limit horizontal movement, making further treatment of horizontal strabismus difficult and potentially causing rotational strabismus [15].

The novelty of our method is that we split and transposed the superior rectus inferiorly into the lateral and medial recti. A suspended sling was created by reinserting the split superior rectus tendons at the maximum stretch point into the sclera. This technique was corrected for 36 PD of hypotropia, similar to the standardized and accepted techniques [2,9,15,16]. Our approach of using a paralyzed muscle has the advantage of using only one muscle. To our knowledge, this has not been described in the literature.

The elasticity of skeletal muscles may have contributed to this result [11,17]. Passive tension develops when a resting skeletal muscle is stretched because the elastic components of the muscle resist stretching. This passive tension is caused by connective tissue and titin proteins in the muscle fibers [18]. Stretching allows thick and thin filaments to glide past each other at the sarcomere level, increasing the overlap between actin- and myosin-binding sites, and permitting the formation of additional cross-bridges. Mechanical studies on the extraocular muscles have confirmed their hyperelastic properties, wherein the stiffness of the extraocular muscles increases more than linearly with increasing elongation [12,18]. The reinsertion point of the paretic muscle along the Tillaux spiral anterior to the equator and close to the lateral and medial rectus insertions may let the mechanical force vectors function in suspending and raising the globe, similar to the arms of a bookshelf.

Although the use of the paralyzed muscle may not lead to an improvement in gaze ability, previous studies on superior rectus resection in MED cases with completely limited supraduction have shown postoperative improvement. This was explained in part by residual force generation within the muscle that may only be detected by electromyography studies but can nevertheless result in an improvement of the upward gaze if strengthened by resection or advancement, as in our case [15].

Our procedure also has several limitations. First, residual hypotropia of >6 PD is universally accepted as a successful outcome of vertical muscle surgery. However, the 9 PD of residual hypotropia is not far from that number, considering that we operated on only one muscle. Second, there was no improvement in the supraduction capacity of the paralytic superior rectus muscles. An additional step to improve it could be previous resection of the superior rectus before splitting and transposing it to evaluate the improvement in supraduction after surgery. The present study is also limited by its nature as a single case study and its relatively short follow-up duration. To comprehensively evaluate the efficacy and safety of an intervention, it is essential to conduct clinical trials with larger sample sizes and longer follow-up periods.

## Conclusions

We present a new surgical technique to correct hypotropia in a young girl after a previous inferior rectus recession. Superior rectus split and vertical transposition prevent complications from operating on multiple muscles. This alternative to the Knapp procedure may be safer when the inferior rectus was already operated on. The final conclusions require rigorous studies that compare outcomes to established procedures through case series with objective measures, randomized trials, and biomechanical investigations. This report proposes further research to fully assess this technique.
